# 7B2 chaperone knockout in APP model mice results in reduced plaque burden

**DOI:** 10.1038/s41598-018-28031-7

**Published:** 2018-06-28

**Authors:** Timothy S. Jarvela, Tasha Womack, Polymnia Georgiou, Todd D. Gould, Jason L. Eriksen, Iris Lindberg

**Affiliations:** 1 Department of Anatomy and Neurobiology, University of Maryland School of Medicine, Baltimore, MD USA; 2 Department of Pharmacology, College of Pharmacy, University of Houston, Houston, TX USA; 3 Department of Psychiatry, University of Maryland School of Medicine, Baltimore, MD USA

## Abstract

Impairment of neuronal proteostasis is a hallmark of Alzheimer’s and other neurodegenerative diseases. However, the underlying molecular mechanisms leading to pathogenic protein aggregation, and the role of secretory chaperone proteins in this process, are poorly understood. We have previously shown that the neural-and endocrine-specific secretory chaperone 7B2 potently blocks *in vitro* fibrillation of Aβ42. To determine whether 7B2 can function as a chaperone *in vivo*, we measured plaque formation and performed behavioral assays in 7B2-deficient mice in an hAPPswe/PS1dE9 Alzheimer’s model mouse background. Surprisingly, immunocytochemical analysis of cortical levels of thioflavin S- and Aβ-reactive plaques showed that APP mice with a partial or complete lack of 7B2 expression exhibited a significantly lower number and burden of thioflavin S-reactive, as well as Aβ-immunoreactive, plaques. However, 7B2 knockout did not affect total brain levels of either soluble or insoluble Aβ. While hAPP model mice performed poorly in the Morris water maze, their brain 7B2 levels did not impact performance. Since 7B2 loss reduced amyloid plaque burden, we conclude that brain 7B2 can impact Aβ disposition in a manner that facilitates plaque formation. These results are reminiscent of prior findings in hAPP model mice lacking the ubiquitous secretory chaperone clusterin.

## Introduction

Protein chaperones play an important role in maintaining neuronal proteostasis and in preventing aberrant protein aggregation within the brain. Perhaps due to its high metabolic activity, the brain appears to be particularly susceptible to proteostatic insult and expresses a variety of chaperone proteins to assist with proper protein handling. Derangement of this process in neurodegenerative disease leads to protein deposition in the form of Lewy bodies (Parkinson’s disease) or amyloid plaques/tau tangles (Alzheimer’s disease and other dementias). Chaperones that have been implicated in neurodegenerative disease include members of the heat shock protein family (reviewed in^1–3^); the ubiquitous secretory chaperone clusterin (reviewed in^4,5^); and a variety of other cytoplasmic and secreted chaperones (reviewed in^6–8^). While many secreted chaperones are clearly active as anti-aggregants *in vitro* (reviewed in^9^), it has been difficult to parse out their actual biological roles during neurodegenerative disease processes. For example, mice lacking total expression of the secreted chaperone clusterin (also known as ApoJ), a known Alzheimer’s risk gene^4,10^, do not exhibit increased but rather reduced numbers of amyloid plaques^11,12^. How brain chaperones handle the increasing burden of protein aggregation, both during normal aging and during the development of neurodegenerative disease, is a topic of increasing interest.

Given the great susceptibility of neurons to proteostatic errors, it might be expected that neurons would express either large quantities of ubiquitous protein chaperones, and/or chaperone proteins specific to neurons. Indeed, many ubiquitous chaperone proteins, such as various heat shock proteins and clusterin, are relatively highly expressed in the brain as compared to other tissues (reviewed in^13,4^). With regard to neuronal-specific chaperones, to our knowledge, the only two chaperone proteins active as potent anti-aggregants *in vitro* whose expression is almost entirely restricted to neurons and endocrine cells are the small secretory proteins known as proSAAS and 7B2^14–16^.

The 7B2 protein is best known as an obligate chaperone for the prohormone convertase proPC2, an enzyme precursor which is totally inactive in the absence of 7B2 due to spontaneous aggregation^17^. 7B2 is synthesized as a 27 kDa precursor that is cleaved into a 21 kDa species within the secretory pathway by the proprotein convertase furin^18^; its anti-aggregant domain, which bears no similarity to any known chaperones, has been localized to an internal, approximately-100 residue segment^16^. Like many chaperones (for example, heat shock proteins^1^), 7B2 is predicted to be primarily unstructured or metastable, and itself forms oligomers^19^. In addition to blocking the aggregation of proPC2, 7B2 also blocks the aggregation of the islet amyloid polypeptide^20^; unfolded IGF-1^21^, and Aβ42^16^, suggesting general anti-aggregant activity. We have put forward the hypothesis that 7B2 functions as a general chaperone to block deleterious self-association of aggregation-prone proteins, both within the regulated secretory pathway and extracellularly^22^. In the experiments described below, we used 7B2 knockout mice to investigate the effect of 7B2 deficiency on plaque formation and cognition in an Alzheimer’s model mouse.

## Results

### 7B2 loss in APPswPS1 mice does not affect memory

**Figure 1:**
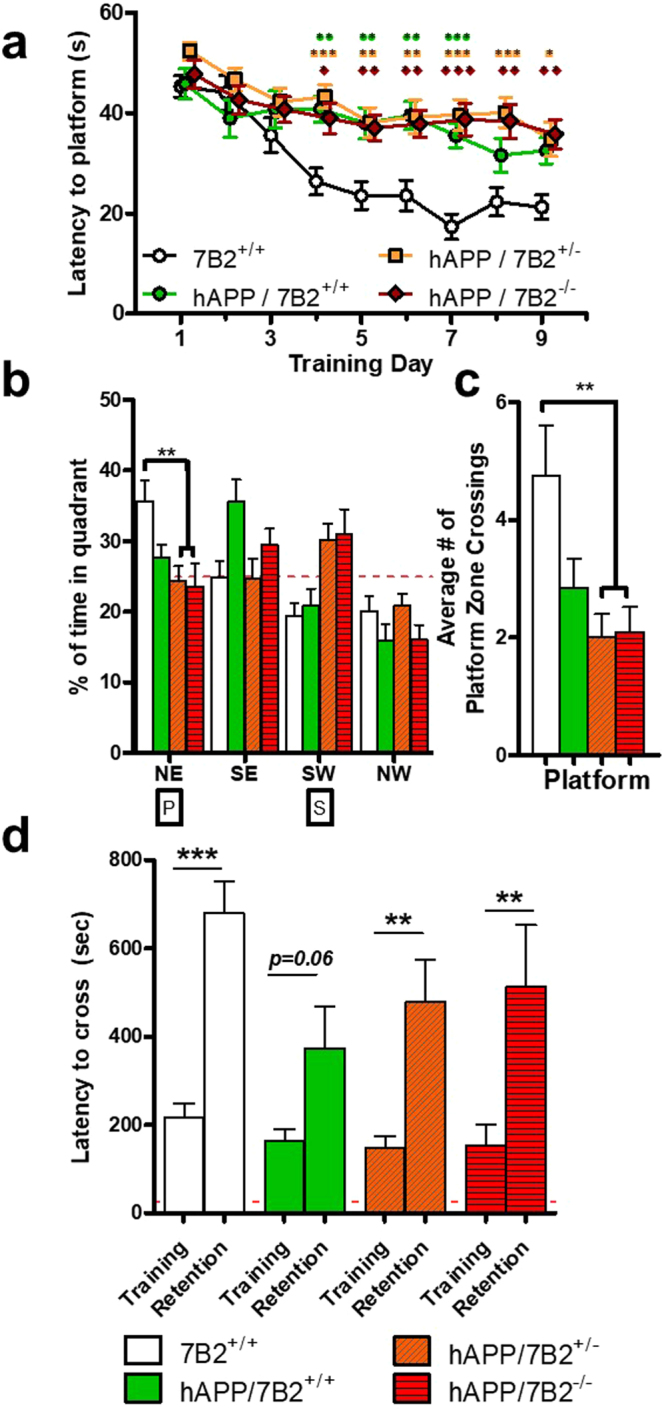
7B2 loss in hAPP transgenic mice does not affect memory. Mice were trained in a genotype-blind manner on a hidden platform Morris water maze for nine days. (**a**) Latency time. The average latency time to platform from Day 1 to Day 9 of training is shown. Mice expressing the hAPP transgene showed significantly longer latency times to platform, but the loss of 7B2 did not cause further increases in latency. Symbols above the line graph represent p-values from two-way ANOVA with Tukey Test for the corresponding genotype. *p < 0.05, **p < 0.01, ***p < 0.001 (**b**) Probe test. One day after training, the mice underwent a single probe test with the platform removed. Wild-type mice lacking the APP transgene spent significantly more time in the NE quadrant, which originally contained the platform, than did hAPP^tg^7B2^+/−^ or hAPP^tg^7B2^−/−^ mice. The red/dashed line denotes 25%, marking equal time in each quadrant. (**c**) Platform crossings. The average number of times each mouse crossed the former platform zone during the 60-sec probe test. hAPP^tg^7B2^+/−^ and hAPP^tg^7B2^−/−^ showed significantly less platform crossings than wild-type mice lacking the hAPP transgene. (**p < 0.01). (**d**) Passive avoidance test. One day after the initial shock training, the latency to cross from a brightly-lit compartment to a dark compartment provides an index of memory retention, with a longer latency representing a stronger memory association. While certain groups showed significant memory retention, no significant differences were found between any 7B2 genotype group. Results are given as the mean ± SEM., n = 12 per group (*p < 0.05; **p < 0.01). No significant differences were found between sexes; thus, all results were pooled.

The Morris water maze test was used to assess spatial navigation cognitive function. Shown in Fig. 1, panel a is the average latency time to platform on training days 1 to 9. Mice expressing the human amyloid precursor protein (hAPP) transgene showed significantly longer latency times to platform, but the loss of 7B2 neither increased nor decreased latency. A 60-second probe test to examine retrieval of memory on the following day yielded similar overall effects of the hAPP transgene that were not influenced by 7B2 genotype. As shown in panel b, mice lacking the hAPP transgene spent significantly more time in the NE quadrant originally containing the platform than did hAPP^tg^7B2^+/−^ or hAPP^tg^7B2^−/−^ mice. The average number of times each mouse crossed the former platform site is shown in panel c. The total platform crosses indicated a trend towards decreased performance in all APP transgene-positive mice (regardless of 7B2 status), with hAPP^tg^7B2^+/−^ and hAPP^tg^7B2^−/−^ showing a significant decrease. In sum, while the averages of all groups indicated learning over the training period, no significant differences were found between 7B2 genotypes.

A passive avoidance test was used to provide a complementary index of memory retention. This test measures the latency to cross from a brightly-lit compartment to a dark compartment, with a longer latency representing a stronger memory association. While most genotypes showed significant memory retention, again, no significant differences were found between the various 7B2 genotypes (Fig. 1, panel d).

### Brain 7B2 levels are not affected by hAPP transgene status; heterozygotes express approximately half as much 7B2 as wild-type mice. Clusterin levels are not impacted by 7B2 genotype

**Figure 2:**
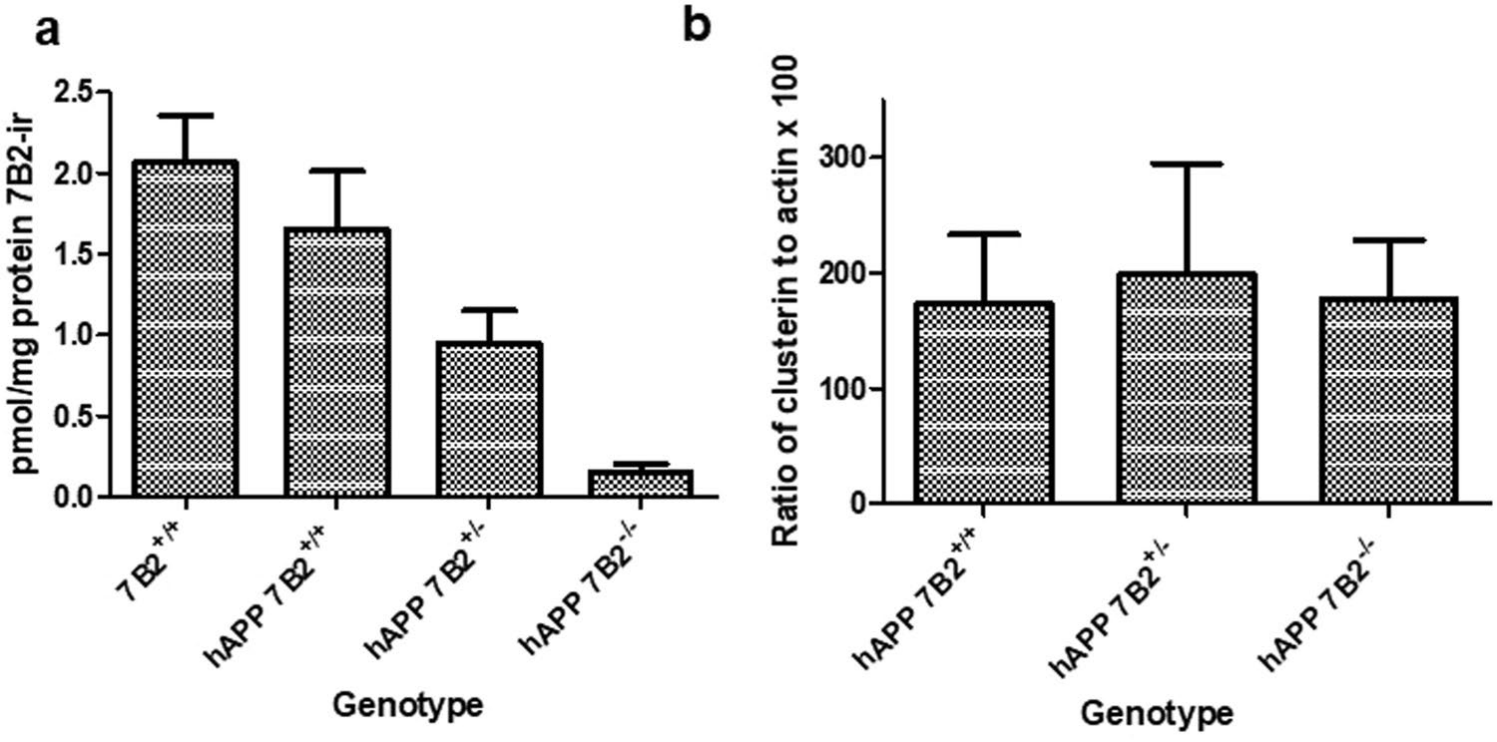
7B2 content does not change as a function of hAPP transgene status; clusterin levels are not altered as a function of 7B2 genotype. Hemibrains were extracted in acid, and aliquots of the clarified supernatants dried and subject to radioimmunoassay for 7B2 (**a**). In (**b**), the ratio of total clusterin to total actin in aliquots of RIPA-buffer extracted hemibrains is shown. Results indicate the mean+/− the SD for 5–8 mice per group in panel (a) and 5 mice per group in panel (b).

Radioimmunoassays (RIAs) for 7B2 were performed in order to confirm genotypes, determine 7B2 expression levels in 7B2 heterozygotes, and investigate a possible effect of hAPP transgene status on brain 7B2 levels. The levels of brain 7B2 (pmol/mg protein) in the various genotypes of 12-month old mice are shown in Fig. 2a. These data confirm the loss of 7B2 expression in 7B2 knockouts; indicate that 7B2 heterozygotes express approximately half as much 7B2 as wild-type mice; and demonstrate no significant effect of hAPP transgene expression on brain 7B2 levels. Figure 2b shows that the level of clusterin, normalized to actin, was also not altered in hAPP mice bearing different 7B2 genotypes.

### 7B2 loss does not impact total brain levels of insoluble and soluble Aβ, or of APP

**Figure 3:**
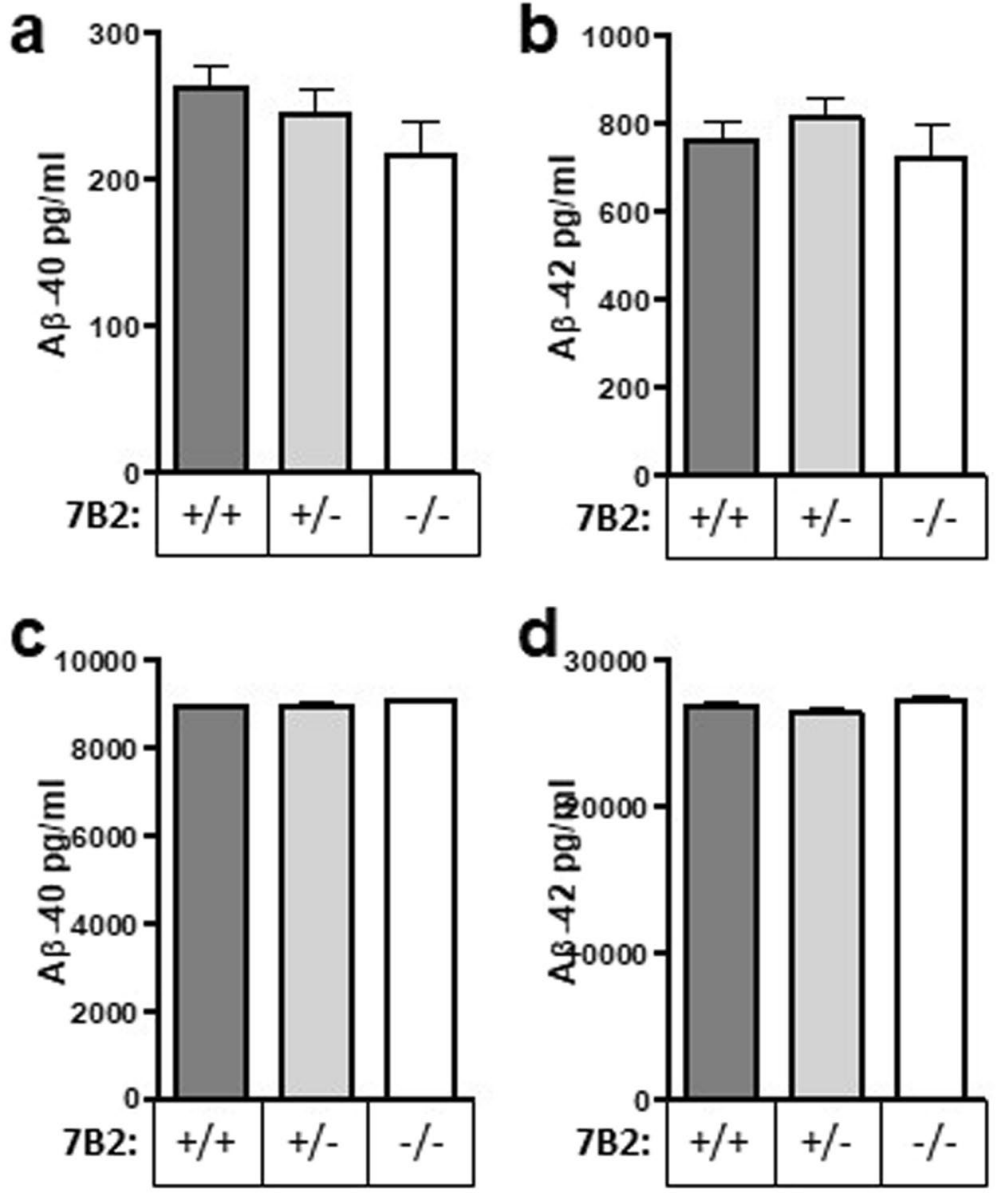
7B2 loss does not impact total brain levels of insoluble and soluble Aβ. Levels of soluble Aβ-40 (**a**) and Aβ-42 (**b**) and insoluble Aβ-40 (**c**) and Aβ-42 (**d**) were assessed by ELISA of RIPA-extracted hemibrain homogenates. Data are shown as the mean ± SD; n = 12 mice per genotype. No significant differences were found between sexes, nor between genotypes.

The soluble and insoluble fractions prepared from brains of various 7B2 genotypes were analyzed using Aβ-40 and −42 human Aβ ELISA kits (Fig. 3, panels a,b and c,d respectively). Wild-type control mice exhibited no human Aβ signal (data not shown). These data demonstrate that 7B2 loss does not significantly impact total brain levels of these two Aβ peptides, either soluble or insoluble.

**Figure 4:**
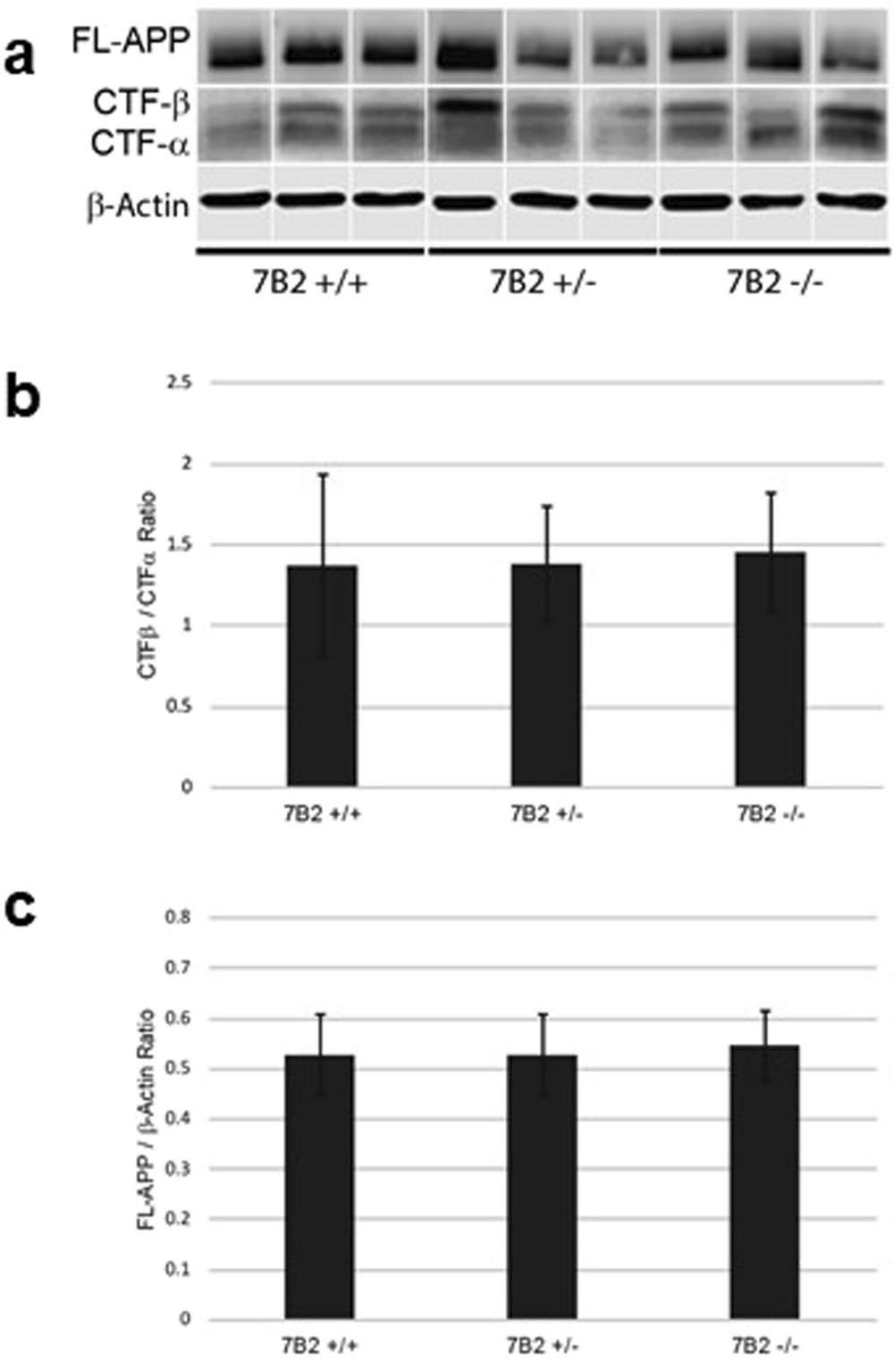
7B2 loss does not alter the level of hAPP in hemibrain homogenates. (**a**) Composite Western blot images show examples of full-length APP, the CTFβ and CTFα fragments, and actin expression in hAPP^tg^ mice of various 7B2 genotypes (7B2^+/+^, 7B2^+/−^ and 7B2^−/−^). Raw corresponding Western blot images can be found in Supplemental Fig. 1. (**b**) FL-APP Analysis. Full-length APP was quantified using densitometric analysis and normalized to the actin loading control. No significant differences between the three genotypes were found. (**c**) CTF Analysis. The ratio between CTFβ and CTFα was compared between the three different genotypes using densitometric analysis. Results depict the mean± the S.E. of 12 mice per genotype. No significant differences were found between sexes, nor between genotypes.

We also investigated the possibility that 7B2 expression might impact total brain levels of APP. However, quantification of Western blots for APP normalized to an actin loading control (Fig. 4, panel a), showed no difference in APP levels between genotype groups (Fig. 4, panel b). (Because Western blotting was performed blind to genotype, lanes were digitally re-grouped. Full-length gels are shown in Supplement 1). Similarly, the loss of 7B2 expression did not alter the ratio of CTFβ to CTFα (Fig. 4, panel c).

### 7B2 loss significantly reduces the number and total area of thioflavin S- and Aβ-immunoreactive plaques

**Figure 5:**
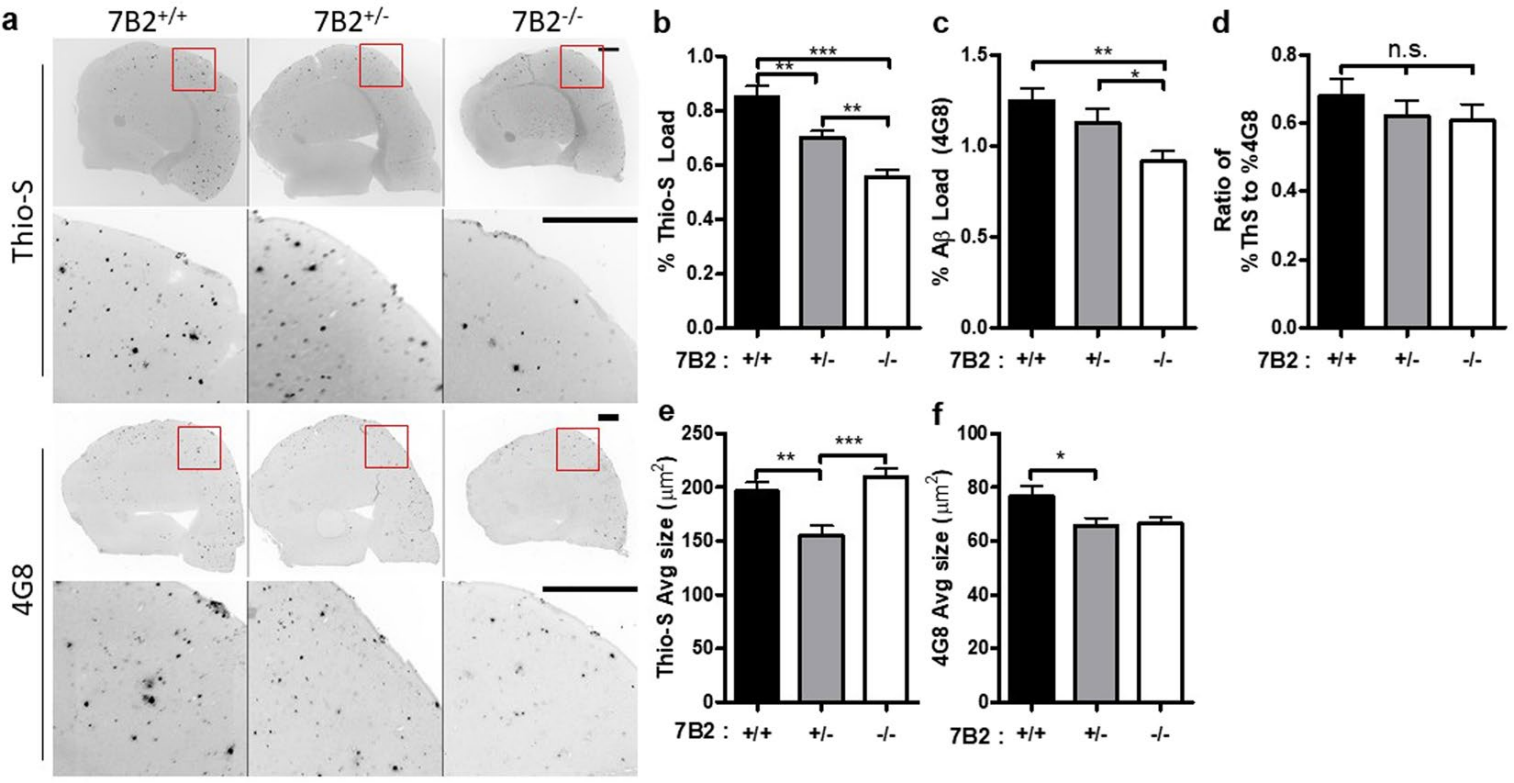
Staining of mouse cortex reveals decreased plaque burden in 7B2 knockout mice. (**a**) Thioflavin-S staining and anti-β-amyloid immunostaining (4G8) were performed on sections of 12-month old mouse cortex expressing the hAPP transgene with the listed 7B2 genotypes. Representative sections from a rostral, dorsal portion of the cortex are shown for each genotype with a small subset (red box) enlarged below. Images are show in inverted contrast, with positive objects as black to enhance visibility. While all mice developed Thio-S and 4G8 positive staining, 7B2^+/−^ and 7B2^−/−^ mice showed a significantly decreased load for each stain as compared to 7B2^+/+^. The percent area of the cortex positive for Thio-S or 4G8 staining was quantified in panels (b) and (c) respectively. The amount of cortical area covered by Thio-S or 4G8 staining was significantly greater in 7B2^+/+^ mice as compared to 7B2^+/−^ and 7B2^−/−^ mice. (**d**) The ratio of the Thio-S load to the 4G8 load showed no significant change between genotypes. (**e**) The average size of Thio-S- and (**f**) 4G8-positive objects. The mean ± SEM, of the total load per slice (panels b and c) and of the average size (panels e and f) is shown. Data were analyzed using a one-way ANOVA, Newman-Keuls test; *p < 0.05, **p < 0.01,***p < *0.001*). Bar = 300 µm.

After the completion of behavioral assays, mice were sacrificed and brains fixed. Hemi-sectioned mouse brains were prepared for immunofluorescence analysis of plaques using either Thio-S staining or immunoreactivity to the 4g8 Aβ antibody. Figure 5a depicts typical brain sections stained with either Thio-S or 4g8 antiserum (using inverted contrast to reveal plaques as dark spots), while Fig. 5b–f presents the quantification of these data. With the loss of 7B2, the overall number of discrete plaques per brain area and the total area of plaques per brain area (percent load), were significantly decreased, both for Thio-S positive plaques (Fig. 5b) and 4G8-positive plaques (Fig. 5c). There was no change in the ratio of Thio-S to 4G8 load, indicating that both β-amyloid deposition and fibril formation of fibrils were equivalently decreased in 7B2^−/−^ mice (Fig. 5d). Panels 5e and f show no significant change in the average object size between 7B2^+/+^ and 7B2^−/−^ mice. In summary, these data show a significant reduction in total observable plaques, as well as in total plaque area per brain, as a consequence of 7B2 loss.

## Discussion

A variety of chaperone proteins contribute to neuroprotection in cellular and animal models of neurodegeneration (for recent reviews, see^7,9,23^). The cytosolic chaperone prefoldin inhibits the aggregation of the huntingtin protein^24^, while the Brichos domain, found in a number of secreted proteins, is able to bind to hydrophobic segments of aggregating Aβ oligomers and block their neurotoxicity^25^. The abundant secretory chaperone clusterin is able to block formation of Aβ fibrils *in vitro*, and Aβ neurotoxicity in cells (reviewed in^5,26^). All of these proteins share the property of being able to bind to exposed hydrophobic regions within malformed proteins. However, the relationship between chaperone expression and neurotoxicity in living animals is not straightforward. While intraventricular injection of Aβ together with clusterin results in improved cognition in spatial navigation tests over Aβ injected alone^27^, genetic knockout of clusterin in two different mouse models of Alzheimer’s results in fewer rather than greater numbers of amyloid plaques, in one case accompanied by reduced neurodystrophy^11,12^. No cognitive studies were reported in these studies, although a recent preliminary report indicated improved cognition in clusterin-deficient 5xFAD hAPP mice relative to hAPP mice expressing normal levels of clusterin^28^. In stark contrast to results obtained with clusterin knockout mice, the expected increase in brain amyloid deposition is observed in mice deficient in the secreted chaperones RAP (receptor-associated protein)^29,30^ and progranulin^31^. These intriguing, apparently contrary animal studies indicate an incomplete understanding of fundamental chaperone mechanisms involved in amyloid deposition.

Like clusterin, the small secretory protein 7B2 is found in association with plaques in cortex obtained from human Alzheimer’s patients, as well as in the cortex of AD model mice^16^. The brain concentrations of 7B2 (this study and^16^) are estimated to be in the near-micromolar range; indeed, given the likely further concentration of peptide at the synapse, synaptic 7B2 concentrations may reach levels many times this. Interestingly, CSF 7B2 concentrations have been reported to decline during normal aging^32^. While in the work reported here we found no change in brain 7B2 levels as a function of APP transgene status, three proteomics studies have identified 7B2 in CSF taken from patients with neurodegenerative diseases involving protein aggregation; in two of these, 7B2 levels were increased in patient CSF^33–35^. A small increase in 7B2 expression in Alzheimer’s cortex, assessed by Western blotting, was also noted in a study of 7 Alzheimer’s patients and 7 controls^36^; however, an earlier study, accomplished using RIA, showed no differences in cortical 7B2 levels between 3 Alzheimer’s patients and 5 controls^37^. These patient numbers are likely too small to form conclusions regarding 7B2 expression as a function of disease in humans.

Our previous work has demonstrated that virally-mediated overexpression of 7B2 is able to block the neurotoxic actions of amyloid peptides added to Neuro2A cells; and, conversely, that siRNA inhibition of endogenous 7B2 production increases Aβ42 neurotoxicity in this system^16^. Thus, 7B2 loss might logically have been predicted to worsen both plaque pathology and cognition in a transgenic APP mouse model. However, the data presented in this report show that similarly to clusterin loss, 7B2 loss reduces plaque formation in the hAPP transgenic mouse brain. While the total observable load of Aβ is decreased with 7B2 loss, only thioflavin-S positive plaques are reduced upon clusterin loss^11^. 7B2 loss appears to have no effect on cognition. We did not observe a significant decrease in soluble Aβ in 7B2 knockouts, as reported in certain clusterin knockout mice^11^; this may explain our lack of change in cognitive performance, since current evidence supports the idea that the level of soluble Aβ correlates more closely with cognitive decline than does the level of insoluble amyloid^38^.

Several alternative hypotheses might explain the beneficial effect of 7B2 loss on amyloid pathology. First, the 7B2 chaperone might normally act within the biosynthetic pathway of APP to promote the production of plaque-forming species; for example, by stabilizing aggregation-prone intermediates within neurons such that an amyloidogenic cleavage pathway is favored. We did not detect increased production of Aβ peptides in total 7B2 knockout brain, arguing against a specific role for 7B2 in favoring amyloidogenic hAPP cleavage. This result is similar to prior findings in a clusterin knockout mouse model which found no change in cleavage of brain hAPP to amyloidogenic species^11^; in another study, reduced rather than increased production of brain Aβ40 and 42 was observed in clusterin-deficient mice^12^. Second, 7B2 expression might negatively impact the degradation of amyloid aggregates, either by hindering their extracellular proteolytic clearance^39^; or, less likely, by blocking amyloid degradation after reuptake into an intracellular degradative compartment. We explored the possibility that 7B2 loss might impact clusterin levels, thus indirectly reducing plaque burden; but no such association was found.

Lastly, reduced Aβ clearance from the brain has been proposed as a major mechanism for clusterin knockout effects. Support for this idea has come both from studies directly confirming reduced Aβ perivascular clearance in clusterin knockout mice expressing hAPP transgenes^12^; and from human studies showing reduced Aβ42 in the CSF taken from Alzheimer’s patients with loss-of-function clusterin alleles^10,40^. However, given increased Aβ retention within the brain, it is still difficult to explain why total plaque levels are so dramatically reduced in clusterin-deficient mice. *In vitro* studies have indicated that clusterin can actually facilitate amyloid aggregation when its molar ratio to Aβ is low^41^; and a recent study has shown that the ratio of clusterin to Aβ varies significantly across brain areas possessing different degrees of plaque pathology^42^. Perhaps 7B2, like clusterin, also participates in an extracellular Aβ sequestration process that becomes pathogenic under conditions of Aβ molar excess.

In conclusion, the 7B2 results reported here extend the paradox of reduced neuropathology in secretory chaperone knockouts in AD model mice. Further work exploring the disposition of amyloid species in chaperone-impaired model mice is needed to shed light on normal mechanisms involved in brain amyloid proteostasis.

## Methods

### Mouse Strains and Handling

C57BL/6J homozygous 7B2 null male mice (7B2^−/−^)^43,44^ were crossed with heterozygous female C57BL/6J APPswe/PS1dE9 transgenic mice (hAPP^tg^) (Jackson Laboratories; Stock No: 034829) to produce F1 heterozygotes. Pairs of F1 mixed heterozygote progeny were then mated to produce an F2 generation containing the four tested genotypes: 7B2 ^+/+^; hAPP^tg^ 7B2^+/+^; hAPP^tg^ 7B2^+/−^; and hAPP^tg^ 7B2^−/−^. Mice were maintained on a normal 12 h light/dark cycle (lights on at 7 am) and provided *ad libitum* access to food and water. Mice were divided into two cohorts, separated by one month, and were maintained for a total of 12 months, with a Morris water maze test at 12 months. A subset of mice was also tested at 12 months with the passive avoidance test. All protocols involving mice were approved by the Institutional Animal Care and Use Committee (IACUC) of University of Maryland School of Medicine in accordance with all guidelines and regulations. Both males and females were used; numbers for each are given below.

### Behavioral Assays

#### Open field test

One week prior to Morris water maze testing, mice were placed in an open field arena of 44 cm × 44 cm × 44 cm for 15 min. Video tracking using Ethovision XT 8.5 (Noldus; Wageningen, The Netherlands) was used to measure locomotor activity in an open arena over a 30 min period in order to confirm that all mice had comparable levels of activity prior to Morris water maze testing. All mice showed comparable levels of movement, as measured by distance traveled and speed in preliminary tests (data not shown).

#### Morris water maze

At 12 months of age, animals were tested in a hidden platform Morris water maze in a 130 cm opaque pool, with a 10 cm platform placed in the northwest quadrant approximately 30 cm from the edge, and 0.5 cm under the surface of the pool, in the center of one of the quadrants. The temperature of the pool was between 21 °C and 22 °C. Mice were tested between 10 am and 6 pm. Distal cues were set up on the walls outside of the pool at 4 cardinal points (North, South, East, and West). Mice were placed into the pool towards the edge of the pool in each quadrant (NE, SE, SW, and NW) and given 60 sec to find the platform. Mice that were unable to find the platform were guided there manually before being removed from the pool. This procedure was repeated for a total of 4 times each day; during this process, mice became trained for release from every quadrant. Mice were trained for a total of 9 days before testing in a probe test. The mice were divided into two cohorts and assayed one month apart. Each group consisted of 12 mice per sex per genotype.

#### Probe test

One day after their last day of training, the platform was removed, and mice were placed into the pool at the southwest quadrant. The mice were tracked for 60 sec and subsequently removed from the pool.

#### Acquisition and analysis

Videos of mice were recorded using a Raspberry Pi computer with a 5-megapixel camera module placed above the arenas. Recording was started prior to placing the mouse in the pool and stopped once the mouse reached the platform or once 60 sec had elapsed. After acquisition, video analysis was performed using Ethovision XT 8.5 and the latency to arrive at the platform (hidden platform portion) or the time in the northwest quadrant (probe test) was measured. Analyses were manually inspected and tracks adjusted to match actual mouse movements when necessary.

#### Passive avoidance test

This paradigm targets the ability of mice to behave in a contrariwise manner regarding their innate preference for dark areas as opposed to bright ones. Shuttle boxes were divided into two compartments separated by a guillotine door, one of which was kept dark while the other remained lit. The floor of the shuttle box consisted of electrified parallel metal bars. During the first phase, the mouse was placed in the brightly-lit area facing away from the entrance to the dark compartment. Upon entrance to the dark compartment, the guillotine door closed and the animal was administered a single inescapable 2 second 0.32 mA electric shock, following which the mouse was returned to its home cage. The animals were returned to the shuttle box 24 h later^45^. Entrance into the dark compartment was detected by infrared sensors; signals from a mouse poking its head through the door were ignored unless the mouse fully entered into the dark compartment. No shock was administered during this phase. The latency to enter the dark compartment was automatically assessed by Coulbourn Instruments software (Graphic State 3.1). The trial was terminated following 15 min if the animal did not cross into the dark compartment. Group sizes consisted of the following: 7B2^+/+^, n = 17; hAPP^tg^7B2^+/+^, n = 8; hAPP^tg^7B2^+/−^, n = 13; hAPP^tg^7B2^−/−^, n = 7. A two-way repeated measures ANOVA was performed for genotype and phase factors. This ANOVA was followed by the Holm-Sidak *post hoc* test.

### 7B2 Radioimmunoassay

Radioimmunoassays (RIAs) for 7B2 were performed in order to confirm genotypes, determine 7B2 expression levels in the various 7B2 genotypes, and investigate a possible effect of APP transgene status on brain 7B2 levels. Frozen hemi-brains were sonicated in 1 ml of ice-cold 0.1 N HCl, frozen and thawed, centrifuged in the cold, and 0.5 ml of the clear supernatant lyophilized. After resolubilization and clarification of the dried extracts in RIA buffer, triplicate samples were subjected to RIA for 7B2 using iodinated peptide 7B2 ^23–39^ and rabbit antiserum 13B6 (1:50,000 final dilution), as previously described^46^. Cross-reaction with the 21 kDa form of 7B2, the major form in brain tissue, was estimated to be approximately 30% using recombinant His-tagged 7B2 as a standard.

### Aβ ELISAs and Western Blotting for Human Amyloid Precursor Protein (hAPP) and clusterin

*ELISAs*- Frozen mouse hemi-brains (excluding the olfactory bulb and cerebellum) were homogenized in RIPA buffer (ThermoFisher, Waltham, MA) (1 ml/00 mg tissue wet weight) containing a complete protease inhibitor cocktail (Amresco, Solon, OH). The homogenate was centrifuged at 100,000 × g for 60 min to separate RIPA-soluble and –insoluble fractions^47–49^. The soluble fraction was aliquoted and stored at −80C until ready for use. The insoluble proteins were extracted using brief sonication in 70% formic acid, followed by ultracentrifugation at 100,000 × g for 1 hour; the sample extracts were stored from at −80C until the time of assay.

Soluble and insoluble fractions were analyzed using Aβ-40 and -42 Human ELISA kits (ThermoFisher Scientific). For analysis of soluble fractions, standard curves of synthetic Aβ peptides were prepared with equivalent quantities of RIPA buffer. Insoluble fractions were neutralized by 1:20 dilution using Tris base buffer, and then further diluted 1:2 using ELISA sample buffer; standard curves of synthetic Aβ peptides were prepared with an equivalent quantity of neutralized buffer/ELISA buffer. Values are expressed in pg/ml. Twelve hAPP^tg^ mice were used for each group, as follows: 7B2^+/+^, 6 males, 6 females; 7B2^+/−^, 7 males, 5 females; and 7B2^−/−^, 6 males, 6 females. Because ANOVA showed no significant differences by sex, data from males and females were pooled for each genotype.

#### Western Blotting

Aliquots of the RIPA-soluble fractions obtained above were brought to an equal protein concentration using the BCA assay (Pierce Biotechnology Inc., Rockford, IL). Samples were prepared for SDS polyacrylamide electrophoresis by the addition of loading buffer (final concentrations, 25 mM Tris-HCl, pH 6.8, 2% SDS, 0.007% bromophenol blue, 4% beta-mercaptoethanol, 10% glycerol) and boiled at 100 C for 5 min. Equal protein amounts were loaded on 4–20% Tris-HCl TGX gels (BioRad Laboratories, Hercules, CA) and transferred to PVDF membranes (BioRad). For analyses of full-length hAPP, C-terminal fragments, and actin, incubations were in 5% milk/0.1% Tween20-TBS with either β-actin antibody (mouse, 1:200, BosterBio, Pleasanton, CA); or with CT20 antibody to detect full-length hAPP and its C-terminal fragments (rabbit, 1:2000, raised against the C-terminal 20 amino acids of hAPP; a kind gift of Dr. Michael Paul Murphy, University of Kentucky, Lexington, KY). Secondary antibodies were horseradish peroxidase (HRP)-conjugated IgG anti-mouse (goat anti-mouse, 1:2000, BosterBio) or anti-rabbit (goat anti-rabbit, 1:10,000; Cell Signaling Technology, Danvers, MA), as appropriate. Clarity enhanced chemiluminescence Western blotting substrate (Pierce Biotechnology Inc., Rockford, IL) was used to visualize HRP activity. Western blots were captured using a cDiGit Blot Scanner (LI-COR Biosciences, Lincoln, NE). Bands were quantified using ImageStudio Software (LI-COR Biosciences, Lincoln, NE). β-Actin was used as an internal loading control.

Western blotting was performed blind to genotype; thus, lanes containing similar genotypes were re-grouped digitally to create the representative image shown in Fig. 4. Full-length blot images are shown in Supplement 1.

Western blotting for clusterin (R&D #RND-AF2747) and actin (Sigma-Aldrich; A2228) was performed using 12–16 ug of protein taken from the above RIPA buffer extracts, diluted 1:10 in reducing Laemmli sample buffer, boiled, and electrophoresed on two 12% TGX Criterion gels (BioRad), followed by semi-dry blotting to nitrocellulose. Primary antisera were used at a 1:7000 dilution (clusterin) or 1:1000 (actin), while HRP-tagged secondary antisera were used at 1:6000 for clusterin (Sigma A5420) and 1:1000 for actin (Biorad 170-6516). Incubations were as described above, and visualization and quantification performed on a BioRad Imager. Full-length blot images are shown in Supplement 2.

### Immunocytochemistry for Thioflavin S Plaque and Aβ Detection

Mouse brains were hemi-sectioned and halves placed directly into 10 ml of Formalin-Free Tissue Fixative (Sigma, St. Louis MO) and stored at 4 °C until prepared for sectioning. To assess the extent of brain neuropathology, paraffin-embedded hemibrains were serially sectioned at 10 µm intervals. Twelve matched slices from each brain were taken from equivalent regions (plates 12–16, ref.^50^), deparaffinized and processed for amyloid detection. For immunohistochemistry, sections were washed in Tris-buffered saline (TBS), blocked with a streptavidin/biotin blocking kit (ThermoFisher Scientific, Waltham, MA), and then incubated in TBS containing 0.5% Tween and 5% donkey serum for 30 min. Tissues were incubated in 4G8 antibody (1:1000; Biolegend) diluted in blocking solution at 4 °C overnight. After washing, sections were incubated in biotinylated secondary antibody (Jackson Immunoresearch; dilution 1:200), followed by incubation with Dylight 549 Streptavidin. Fibrillar Aβ deposits were visualized using Thioflavin S (Thio-S), following previously described methods^51^. Briefly, mouse brain sections were washed with TBS and stained for 10 min with a solution of 0.5% Thio-S in 50% ethanol. Finally, sections were washed in 50% ethanol and TBS, dried, and coverslipped using Vectashield (Vector Laboratories, Burlingame, CA).

### Quantitative Image Analysis

Stained sections were digitally captured on an Olympus DSU-IX81 Spinning Disc Confocal using a 10X objective, under standardized conditions for each fluorophore using Microbrightfield Software; approximately 300 images per slide were captured. Section data were loaded into the FIJI program^52^, background corrected, and then optically stitched using the Stitching Plugin^53^. The cortex was outlined for each section, and particle analysis was conducted for each region. The results were normalized to the total slice area analyzed. The total Thio-S positive area or 4G8-positive area was analyzed as a percent of total cortex for each individual slice. The means for each slice were then averaged together to give an average % Thio-S and 4G8 load. We analyzed 10–12 sections from 17/23 mice and 6–9 sections from the remaining mice. For the analysis of plaque burden and average size, only objects larger than 5 pixels (2.37 μm^2^) and less than 1500 μm^2^ were included in the analyses in order to reduce artifact.

Graph preparation and statistical analysis was performed using GraphPad Prism 5 (GraphPad Software, Inc). A one-way or two-way ANOVA, with a Tukey *post-hoc* test, was performed, as indicated in the figure legends.

The datasets generated during and/or analyzed during the current study are available from the corresponding author on reasonable request.

## Electronic supplementary material

Full Western Blots
