# Supplementary material for: 7B2 chaperone knockout in APP model mice results in reduced plaque burden
Source: Sci Rep. 2018 Jun 28;8:9813. doi: 10.1038/s41598-018-28031-7 (PMC6023903; doi:10.1038/s41598-018-28031-7)
Supplement: Supplementary file 1 — Full Western Blots [file 41598_2018_28031_MOESM1_ESM.docx]

**7B2 chaperone knockout in APP model mice results in reduced plaque burden**

Timothy S. Jarvela^1^, Tasha Womack^2^, Polymnia Georgiou^3^, Todd Gould^3^,

Jason L. Eriksen^2^ and Iris Lindberg^1*^

^1^Dept. of Anatomy and Neurobiology, University of Maryland School of Medicine, Baltimore, MD

^2^Dept. of Pharmacology, College of Pharmacy, University of Houston, Houston, TX

^3^Dept. of Psychiatry, University of Maryland School of Medicine, Baltimore, MD

# **Supplementary Figure 1**

This figure shows the full-length blots used for the analysis of Figure 3; the genotype below is the 7B2 status of hAPP positive mice. *nTg*; wild-type mouse.


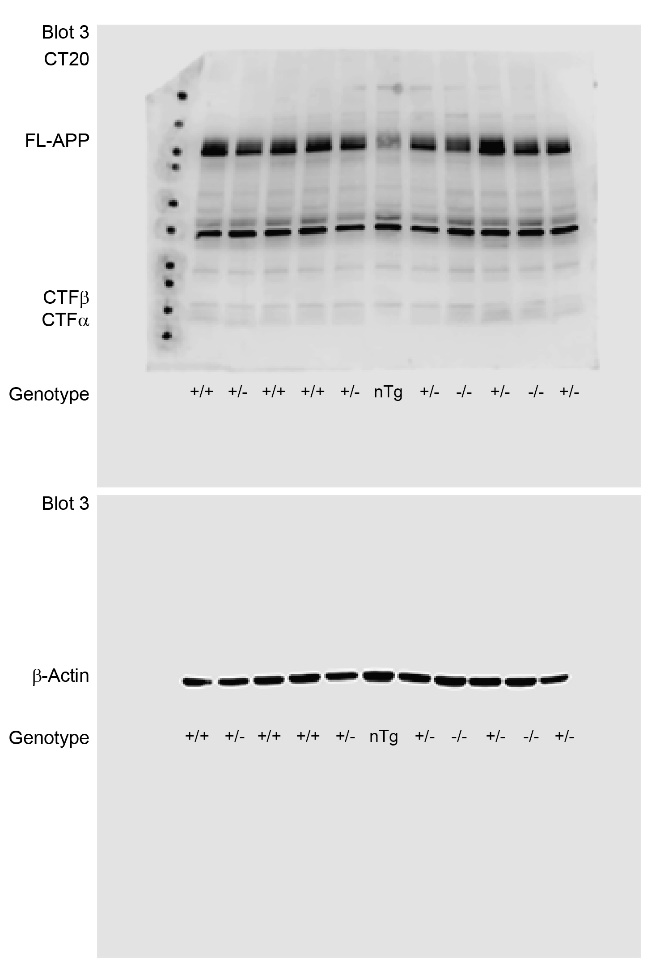

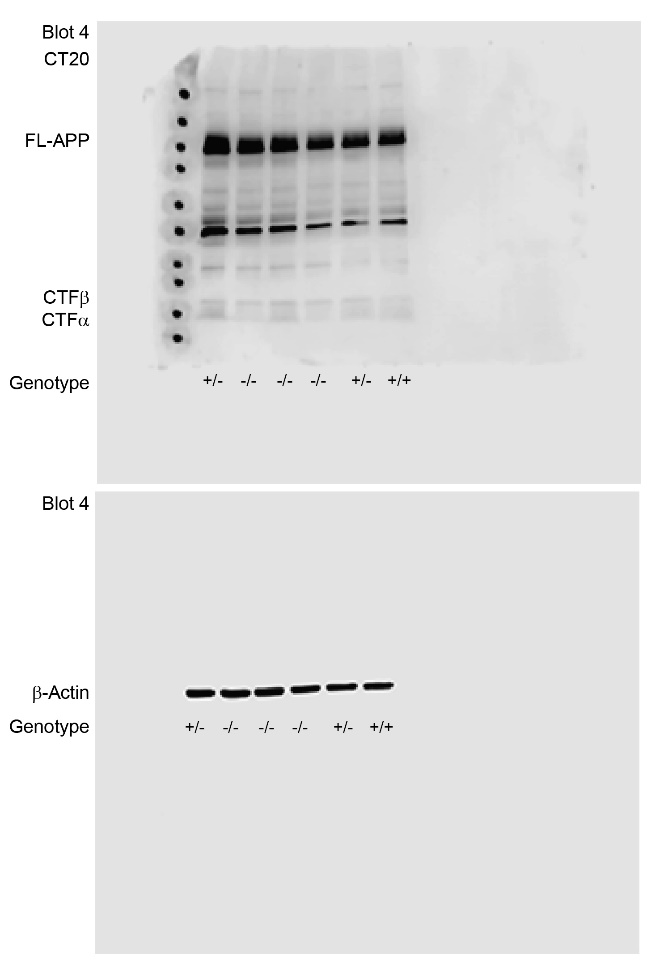

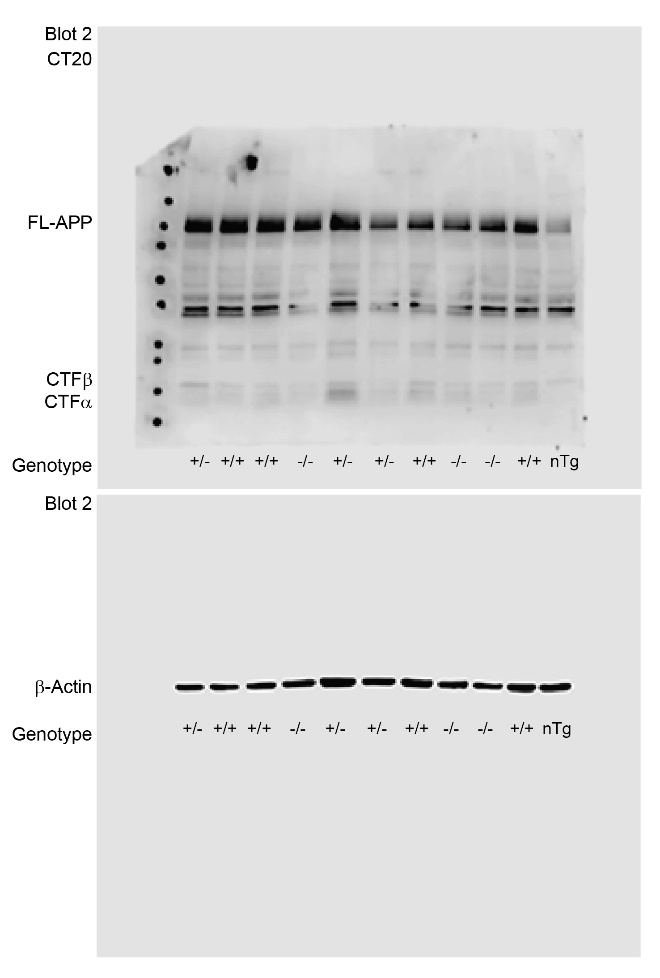

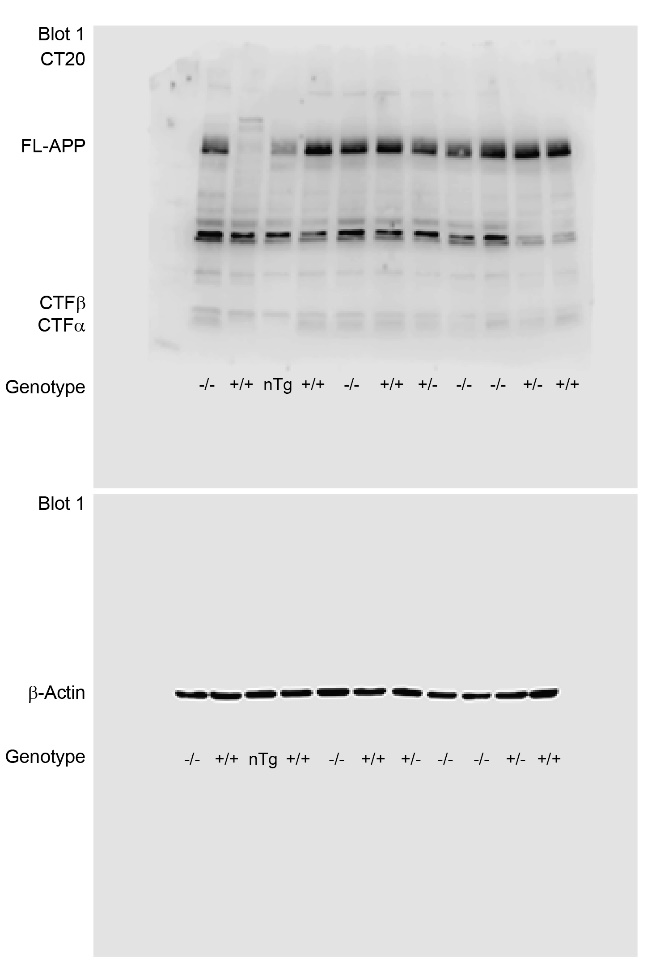


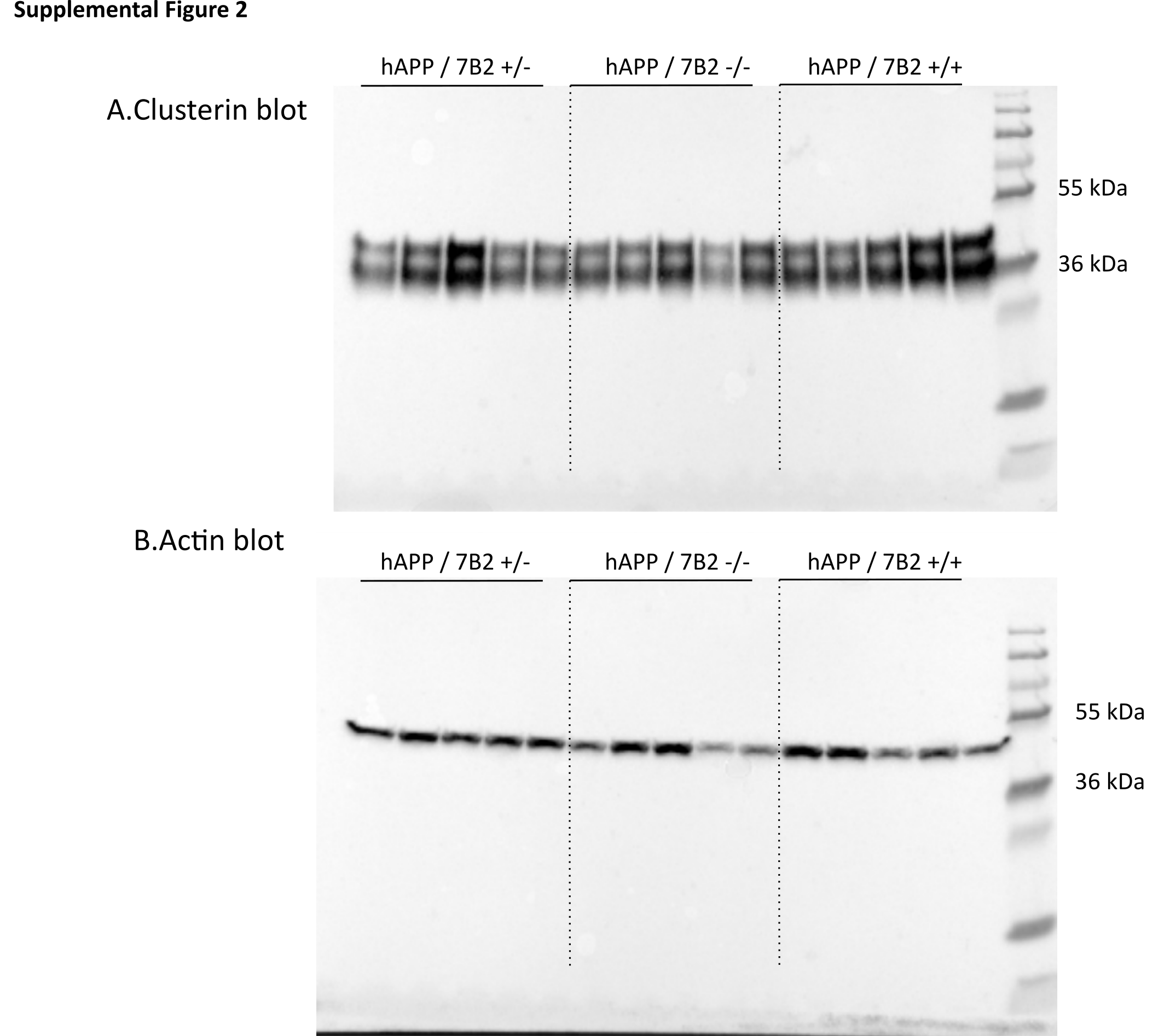


**Supplemental Figure 2. Clusterin levels do not vary due to 7B2 loss.** Western blot images for clusterin (A) and actin (B) that were analyzed in Figure 2B. Genotypes are marked above the lanes.
